# Mechanisms underlying the involvement of peritoneal macrophages in the pathogenesis and novel therapeutic strategies for dialysis-induced peritoneal fibrosis

**DOI:** 10.3389/fimmu.2024.1507265

**Published:** 2024-12-19

**Authors:** Yangwei Wang, Yixian Zhang, Mingqi Ma, Xiaohua Zhuang, Yue Lu, Lining Miao, Xuehong Lu, Yingchun Cui, Wenpeng Cui

**Affiliations:** Department of Nephrology, Second Hospital of Jilin University, Changchun, China

**Keywords:** peritoneal dialysis, peritoneal fibrosis, macrophage, polarization, therapeutic strategies

## Abstract

Long-term exposure of the peritoneum to peritoneal dialysate results in pathophysiological changes in the anatomical organization of the peritoneum and progressive development of peritoneal fibrosis. This leads to a decline in peritoneal function and ultrafiltration failure, ultimately necessitating the discontinuation of peritoneal dialysis, severely limiting the potential for long-term maintenance. Additionally, encapsulating peritoneal sclerosis, a serious consequence of peritoneal fibrosis, resulting in patients discontinuing PD and significant mortality. The causes and mechanisms underlying peritoneal fibrosis in patients undergoing peritoneal dialysis remain unknown, with no definitive treatment available. However, abnormal activation of the immune system appears to be involved in altering the structure of the peritoneum and promoting fibrotic changes. Macrophage infiltration and polarization are key contributors to pathological injury within the peritoneum, showing a strong correlation with the epithelial-to-mesenchymal transition of mesothelial cells and driving the process of fibrosis. This article discusses the role and mechanisms underlying macrophage activation-induced peritoneal fibrosis resulting from PD by analyzing relevant literature from the past decade and provides an overview of recent therapeutic approaches targeting macrophages to treat this condition.

## Introduction

Renal replacement therapy for patients with end-stage renal disease includes peritoneal dialysis (PD), hemodialysis, and kidney transplantation. The efficacy of PD is regarded as comparable to that of hemodialysis in the management of end-stage renal disease (ESRD) (1). Compared to hemodialysis, PD offers several advantages, including a streamlined operational process, the convenience of home-based treatment, and a reduction in hospital visits. This enables patients to allocate more time for professional and academic endeavors while concurrently enhancing their overall quality of life (2). Research indicates that PD can effectively preserve residual renal function and may result in lower treatment costs compared to hemodialysis (3). Findings from a comprehensive study suggest that PD provides a survival advantage during the initial 2-3 years of therapy (2). However, despite these benefits, global uptake of peritoneal dialysis remains suboptimal, with only 10% to 20% of ESRD patients receiving this modality (4). The sustainability of long-term peritoneal dialysis maintenance is constrained by the deterioration of peritoneal function resulting from prolonged exposure to peritoneal dialysate. Prolonged PD induces pathophysiological alterations in the peritoneal architecture, notably progressive peritoneal fibrosis, characterized by enhanced collagen deposition, fibroblast proliferation, and inflammatory cell infiltration within the subcutaneous tissue (5, 6). Specifically, peritoneal fibrosis can result in diminished peritoneal function and ultrafiltration failure, ultimately necessitating the cessation of PD (7). Encapsulating peritoneal sclerosis is a serious outcome of peritoneal fibrosis and the most serious complication of PD, often leading to patients discontinuing PD and significant mortality (8, 9). This condition prone to causing persistent or recurrent intestinal obstruction (10), which is an indication for emergency discontinuation of PD with a mortality rate of up to 50% within one year of diagnosis (9).

The cause and mechanism of peritoneal fibrosis in PD patients are not clear, and no specific treatment is available. However, research points to chronic inflammation of the peritoneum as an underlying mechanism of PD (11). Peritoneal injury due to chronic peritoneal inflammation is thought to be caused by uremia, peritonitis attack, catheter irritation, high glucose concentration, glucose degradation products, low pH, and high osmotic pressure of non-physiological peritoneal dialysate (8, 12). In high glucose environments, production of glucose degradation products of peritoneal dialysate through heat sterilization and low pH is a known independent risk factor for peritoneal fibrosis and angiogenesis (13). At present, to improve the biocompatibility of peritoneal dialysate, neutral pH and low glucose products have been developed that will minimize membrane damage (14). However, recent studies have shown that the application of biocompatible peritoneal dialysate may also cause changes in peritoneal structure, suggesting that biocompatible peritoneal dialysate can only partially reduce peritoneal fibrosis (15).

The aberrant activation of the immune system appears to contribute to alterations in peritoneal structure and peritoneal fibrosis that leads to PD treatment failure, with the overactivation of macrophages as a main cause of peritoneal pathological injury (16, 17). In the subsequent sections, we will elucidate the role and mechanisms of macrophage activation in peritoneal fibrosis induced by PD, as highlighted by recent research findings.

## Macrophage activation

Macrophages are extensively distributed immune cells that serve as a crucial link between innate and adaptive immunity, playing a vital role in the maintenance of homeostasis (18). Macrophages can be classified into two primary categories: monocyte-derived macrophages, which originate from hematopoietic stem cells in the bone marrow and are transported via the bloodstream to sites of inflammation to engage in pathological processes, and tissue-resident macrophages, derived from yolk sac progenitor cells, whose principal role is to uphold homeostasis (19). In various tissue and organ contexts, macrophages exhibit distinct functions and are referred to by specific names such as pulmonary macrophages in the lungs, microglia in the nervous system, osteoclasts in bone, and Kupffer cells in the liver (20). Macrophages play a pivotal role in various processes of the immune system, facilitating the clearance of pathogens, apoptotic cells, and cellular debris. They secrete an array of signaling proteins and modulate adaptive immune responses through antigen processing and presentation (21–23). In addition, macrophages are actively involved in tissue inflammation and damage repair (24, 25). Morphological heterogeneity and functional diversity of macrophages are essentially dependent on their activation state or response to microenvironmental stimuli (26). Macrophages exhibit a high degree of plasticity, enabling them to adapt to environmental influences and specific pathological processes, they can change their phenotype and function. This process of phenotypic change is called macrophage polarization.

**Figure 1:**
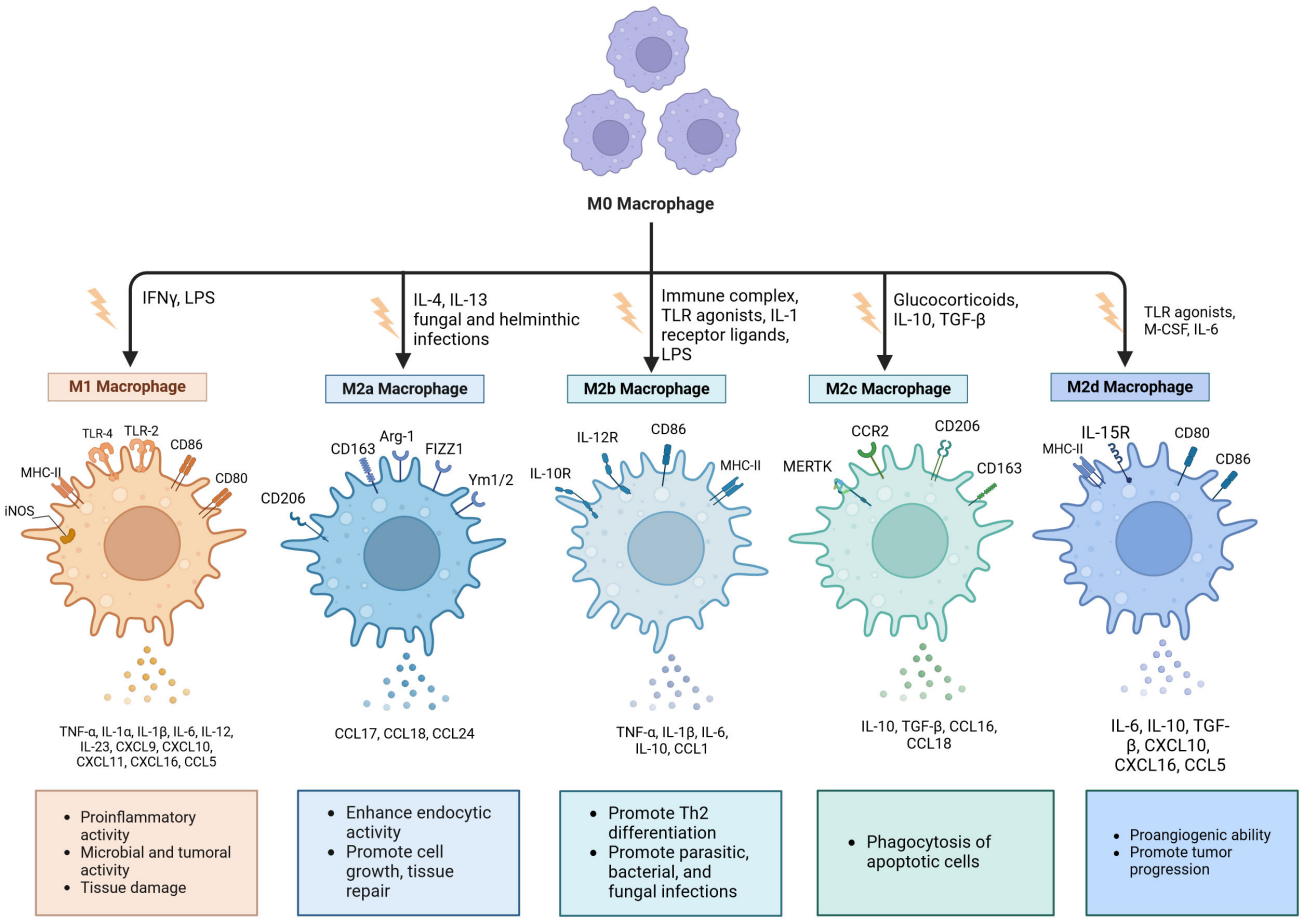
Macrophages undergo polarization into distinct phenotypes. Classically activated macrophages (M1) are generally recognized as pro-inflammatory phenotypes, while selectively- activated macrophages (M2) typically assume an anti-inflammatory/pro-fibrotic role. Based on different activation pathways, M2 macrophages can be categorized into four distinct subtypes: M2a, M2b, M2c, and M2d. Each subtype expresses unique surface markers and performs specific functions. es. Created with BioRender.com (accessed on April 8th, 2024).

The polarization of macrophages is typically characterized by two distinct phenotypes: classically activated macrophages (M1) and alternatively activated macrophages (M2) (27). Interferon-γ (IFN-γ) and tumor necrosis factor-α (TNF-α), both classified as Th1 cytokines, in conjunction with the bacterial product lipopolysaccharide (LPS) and viral infections, promote and enhance the polarization of M1 macrophages (**Figure 1**). Activated M1 macrophages exhibit the expression of Toll-like receptors (TLR) 2 and 4, as well as cell surface molecules including CD80, CD86, inducible nitric oxide synthase (iNOS), and major histocompatibility complex class II (MHCII) (28). They are also capable of producing a substantial array of antigen-presenting and pro-inflammatory cytokines, including interleukin (IL)-1β, IL-6, IL-12, IL-23, TNF-α, CCL2, CCL5, CXCL1-3, CXCL5, and CXCL8-10. Additionally, they express nitric oxide (NO), which induces macrophages to further polarize in a feedback loop while playing both pro-inflammatory and anti-tumor roles (29, 30).

Research has indicated that the LPS/TLR4 signaling pathway plays a crucial role in the polarization of M1 macrophages. This pathway triggers the activation of nuclear factor κB (NF-κB) and interferon regulatory factor 3 (IRF3), leading to the production of pro-inflammatory cytokines such as IL-6 and TNF-α, which contribute to inflammation in various tissues and organs (31). M1 macrophages, which are proinflammatory in nature, play a vital role in clearing pathogens and defending the host. However, when M1 macrophages remain activated for extended periods, they can subject body tissues to a state of chronic inflammation, resulting in tissue injury. To alleviate this prolonged inflammatory response linked to sustained M1 activation, there is a shift towards M2 macrophage polarization, which has notable anti-inflammatory properties (32). Furthermore, some studies have shown that M1 macrophages can also directly or indirectly induce production of matrix metalloproteinases (MMPs) and anti-fibrotic cytokine CXCL10 to promote degradation of the lung tissue matrix, inhibit fibrosis, and exert anti-fibrotic properties (33).

M2 macrophages are a type of alternatively activated macrophage that is triggered by IL-4, IL-10, IL-13, and transforming growth factor beta (TGF-β) (34). Human M2 macrophages are identified by markers such as the mannose receptor (CD206) and CD163, whereas murine macrophages are distinguished by the expression of arginase-1 (Arg1) along with chitinase-like proteins Ym-1 and Ym-2 (35). Activated M2 macrophages are capable of expressing and releasing a range of chemokines and cytokines, such as IL-1RA, IL-10, CCL16, CCL17, CCL18, CCL22, and CCL24 (36).

Based on the various activation pathways involved, M2 macrophages can be categorized into four distinct subtypes: M2a, M2b, M2c, and M2d (**Figure 1**) (37). M2a phenotype macrophages can be stimulated by IL-4, IL-13, and infections caused by fungi and helminths to produce Arg1, CD206, and CD163. Additionally, they release CCL17, CCL18, and CCL24 while being essential for tissue repair processes (38, 39). The M2b subpopulation is activated by ligands of the IL-1 receptor, immune complexes, TLR agonists, and LPS. Following stimulation, this subtype expresses CD86 and MHC-II while markedly enhancing the production of several cytokines including TNF-α, IL-1β, IL-6, and IL-10 (18, 27). The appearance of M2c cells is known as acquired deactivation of macrophages, because this macrophage subset cannot achieve M1 polarization (24). IL-10, TGF-β1, and glucocorticoids induce the M2c subset, which highly express CD206, CCR2, CD163, and MERTK. This subset demonstrates strong anti-inflammatory effects and promotes tissue remodeling through the secretion of elevated levels of IL-10 and TGF-β (32, 40). M2d macrophages are stimulated by IL-6, macrophage colony-stimulating factor (M-CSF), and TLR agonists (27). They exhibit high levels of CD80, CD86, MHC-II, and IL-15 expression while secreting cytokines such as IL-6, IL-10, TGF-β, CXCL10, CXCL16, and CCL5 (18, 36). Studies have shown that M2d cells can promote angiogenesis, adaptive immunosuppression, and matrix remodeling (21, 27). In summary, M2 macrophages are involved not only in the removal of damaged and apoptotic cells along with other debris but also in facilitating tissue regeneration and vascular repair (41).

## Macrophages and PD

Repeated fluid exchange in PD, dialysates with high-glucose concentrations, and PD-associated peritonitis disturb the peritoneal tissue homeostatic state and modify the immune cell makeup within the peritoneal cavity, which are the main causes of technical failure and death of patients (42–44). Recent studies have shown that repeated bouts of bacteria-induced peritoneal inflammation initiate peritoneal immune-mediated repair of damaged tissues, ultimately leading to peritoneal fibrosis (45). These findings indicate that peritoneal immune cells play a role in the development of peritoneal inflammation and fibrosis in patients undergoing PD, with alterations in the immune cell composition within the peritoneum potentially linked to negative outcomes.

Macrophages are key cells in local immune responses, playing roles in pathogen clearance, antigen presentation, and tissue repair (46). *In vivo* studies in different tissues have shown that different macrophage subpopulations have different distributions and functional roles in maintaining tissue homeostasis and responding to local inflammation (47). In the late 1970s, scientists-initiated investigations into the cellular makeup of dialysis effluents from non-infected PD patients and discovered that macrophages were the predominant cell type present in these effluents (48, 49). In 1999, Humphries and colleagues successfully extracted a significant quantity of macrophages from peritoneal dialysis effluents. A single bag of dialysate contained as many as 10 million macrophages, among which macrophages accounted for 57–61%, lymphocytes for 30–41%, mesenchymal cells for 1–6%, and polymorphic cells for <1–3% (50). Several other studies analyzing cells in PD effluents have confirmed that mononuclear/macrophage cells account for about 40% of peritoneal white blood cells in the dialysis effluents of PD patients (51, 52). In 2017, flow cytometry was used to analyze dialysis effluents from 30 PD patients without signs and symptoms of infection or inflammation. Peritoneal leucocytes were found to comprise 44% monocytes/macrophages, 29% neutrophils, 12% T cells, 10% dendritic cells (DCs), and 5% B cells (53). In conclusion, macrophages are the predominant cell type in the effluents of peritoneal dialysis patients.

The researchers subsequently investigated the subtypes of macrophage differentiation in peritoneal dialysis effluents from both human and mouse subjects. *In vitro* studies revealed that the M1 and M2 macrophages derived from the abdominal cavities of both human and mouse display distinct phenotypic features. Further analysis of cytokines expressed by the two macrophage subtypes revealed that M1 macrophages from both human and mouse abdominal tissues exhibited elevated levels of TNF-α, IL-1, and IL-6. In contrast, M2 macrophages showed increased expression of IL-10 and mannose receptor while displaying reduced levels of inflammatory cytokines. Moreover, with respect to the T cell co-stimulatory molecules CD86 and MHC-II, peritoneal M1 macrophages from both humans and mice exhibited significantly elevated expression levels, whereas M2 macrophages demonstrated a markedly reduced expression of CD86 and MHC-II (53). High glucose environments can affect the polarization and function of macrophages. A study examining the impact of elevated glucose levels on both internal and external polarization of peritoneal macrophages found that the proportion of M2 macrophages in the 2.5% glucose dialysate group was significantly greater than that in the 1.5% glucose dialysate group. However, no significant difference was observed in M1 macrophage levels between the two glucose concentrations. Additionally, within a medium containing 138.8 mmol/l glucose, there was an increase in the percentage of M1 macrophages corresponding to longer exposure times (54).

In addition to the presence of M1 and M2 macrophages, other distinct phenotypes of macrophages have been identified in peritoneal dialysis effluents. Taylor et al. (55) employed flow cytometry combined with global transcriptome analysis to investigate the phenotypic traits of CD14+ macrophages and CD1c+ dendritic cells (DCs) present in peritoneal dialysis effluent. In terms of phenotype, CD14+ cells exhibited elevated levels of CD11b, CD16 (FcγRIII), and CD163, while the CD1c+ population demonstrated increased expression of CD11c, Fc 3 R1a, and CD206. Importantly, regarding the characteristics of transcriptional profiles, the CD1c+ subset exhibited significantly elevated expression levels of Fms-related tyrosine kinase 3(FLT3) and interferon regulatory factor 4 (IRF4), while the CD14+ subset demonstrated increased expression of MAFB (V-maf musculoaponeurotic fibrosarcoma oncogene homolog B), which is implicated in macrophage differentiation. These data indicate that the CD14+ subgroup has the transcriptional profile of “traditional MØs”. In terms of function, CD14+ MØs showed a higher phagocytosis capacity and produced more reactive oxygen species than CD1c+ DCs. While both cell types demonstrated effective antigen processing and presentation, CD1c+ dendritic cells exhibited superior efficacy compared to CD14+ macrophages in this context. According to the expression profiles of CD16 and CD206, CD14+ macrophages can be divided into four unique subtypes: CD16+CD206+, CD16+CD206–, CD16–CD206+, and CD16–CD206–. These various subtypes might indicate distinct stages of macrophage activation and maturation within the abdominal cavity (55).

Recent research in macrophage biology has classified these cells into tissue-resident macrophages (MØres), which exist in tissues under homeostatic conditions, and monocyte-derived macrophages (MØmono), which are attracted to the tissue in response to inflammation (56). Importantly, when tissue inflammation occurs, the number of MØres decreases or even disappears, while MØmono are recruited to the site of inflammation (57). Six hours after a single intraperitoneal injection of peritoneal dialysate in mice, the quantity of F4/80 high MØres in peritoneal dialysate effluents decreased to 30% of the original number (58). Of note, the count of F4/80 low MHC-II high MØmono also decreased by roughly 40% when exposed to peritoneal dialysate. Following repeated PD fluid injections, the proportion of MØres in the peritoneal cavity further decreased, while the percentage of Ly6C high monocytes and F4/80 low monocyte-derived MØmono rose. Due to the absence of functional markers like Tim4, CD73, and Vsig4, MØres have diminished their functional capabilities, especially those associated with anti-inflammatory responses and efferocytosis (58). In fact, MØmonos activated by LPSs *in vitro* are also commonly referred to as M1 macrophages (17). IL-4-activated M2 macrophages are distinguished by the presence of CD206, Arg1, and Ym1, and these markers are differently expressed on MØres and MØmono (59, 60). Therefore, the observed function of M2 macrophage activation *in vitro* might merely indicate an increased influx of MØmono, rather than being a result of IL-4-mediated activation (61).

## Polarization of macrophages in PD-induced peritoneal fibrosis

Peritoneal fibrosis continues to pose a major difficulty in managing peritoneal dialysis for individuals with end-stage renal disease. Extensive fibrosis of the visceral peritoneum results in encapsulating peritoneal sclerosis, a serious complication associated with a high risk of mortality (11). With prolonged exposure to elevated levels of glucose and its degradation products, the renin-angiotensin-aldosterone system becomes activated, leading to the production of several pro-inflammatory and angiogenic factors such as NO, TGF-β, and vascular endothelial growth factor (VEGF). These factors lead to peritoneal inflammation and capillary angiogenesis (5, 6). This ongoing inflammation promotes the epithelial-mesenchymal transformation (EMT) or mesenchymal transformation (MMT) in peritoneal mesothelial cells, which leads to mesothelial detachment, mesothelial subfibrosis, and impaired vascular permeability (62, 63).

Earlier research has demonstrated a significant relationship between macrophages and the EMT of peritoneal mesothelial cells. Shi et al. (64) directly co-cultured M1 macrophages and HMrSV5 cells to induce phenotypic changes such as cell elongation, branching, and disappearance of their pebble-like shape. Moreover, the co-cultured HMrSV5 cells exhibited a decrease in E-cadherin levels and an increase in α-SMA expression, indicating that M1 macrophages may facilitate EMT in peritoneal mesothelial cells(PMCs). Conversely, PMCs co-cultured with M2 macrophages displayed no substantial changes. In contrast, the findings of another experimental study produced divergent outcomes. To observe the influence of different subtypes of macrophages on EMT occurrence of PMCs, researchers have co-cultured PMCs with polarized macrophages M1, M2a, and M2c. The results showed that decreased expression of E-cadherin and increased α-SMA expression were observed in PMCs co-cultured with the three macrophage subtypes. In PMCs co-cultured with M2c, these two activation characteristics were more obvious, indicating that M2c macrophage polarization can enhance the EMT of PMCs, while M1 and M2a polarization may have a more limited effect than M2c (65).

Glucose serves as the primary osmotic agent in most PD solutions, and prolonged exposure to elevated glucose levels is a significant factor contributing to peritoneal fibrosis. In this process, macrophages, as the main inflammatory cells, are stimulated by various cytokines and polarized into different phenotypes, including a pro-inflammatory M1 macrophage phenotype and anti-inflammatory M2 macrophage phenotype (55), that collectively contribute to the development of chronic peritoneal inflammation and fibrosis in PD.

High glucose levels promote the polarization of peritoneal macrophages into an M2 phenotype, which may be crucial in the development of fibrosis associated with PD (54). Experimental animal studies have shown that the subcutaneous interperitoneal layer in continuous PD rats is fibrotic with neovascularization, and macrophages with M2 characteristics accumulate in the fibroblast area (66). In humans, polarized M2 macrophages might contribute to peritoneal fibrosis by promoting the growth of fibroblasts (67). In C57BL/6J mice, peritoneal fibrosis is triggered by the intraperitoneal administration of hyperglycemic dialysate, and peritoneal injection of clodronate sodium (LC, a macrophage-specific scavagant) is used to treat peritoneal fibrosis in mouse models. This demonstrated that in the mouse peritoneal fibrosis context, there was an upregulation of peritoneal thickness, and the expression levels of Col-1, fibronectin, CD206, TGF-β, y-1, and Arg-1. In contrast, these markers were downregulated in the LC treatment group. These findings suggest that M2 macrophages in the peritoneum play a significant role in PD-related peritoneal fibrosis and could serve as a potential therapeutic target for this condition (68).

Nonetheless, research has indicated that the polarization of M1 macrophages is also implicated in peritoneal fibrosis induced by PD. PD markedly elevated the levels of TGF-β1, VEGF, and ALK5 proteins in peritoneal tissue, along with increasing α-SMA expression, which is a marker for peritoneal fibrosis and fibroblasts. Additionally, it significantly reduced the expression of activation-related proteins in M2 macrophages (Erg2, IRF4). Nevertheless, the levels of activation-related proteins in M1 macrophages (CD38, IRF5) were not significantly impacted (69). *In vivo*, after 5 weeks of catheterization with high-glucose PD fluid, peritoneal PKCβ was upregulated, promoting pro-inflammatory M1 polarization and PKCα upregulation, and the levels of IL-6, TNF-α, and monocyte chemotactic protein-1 (MCP-1) were increased, leading to an inflammatory response and peritoneal injury, manifested by fibrosis and neovascularization (70). However, it has been demonstrated that M1 macrophages facilitate the process of EMT in PMCs, necessitating direct cell-to-cell contact rather than reliance on macrophage-secreted cytokines for their functional effects (64). In mouse models where macrophages were depleted through abdominal dialysis, there was a notable decrease in α-SMA and fibronectin expression, along with an increase in E-cadherin levels, indicating a general reduction of peritoneal fibrosis in these macrophage-depleted mice. Following the reperfusion of M1 macrophages, both structural and functional damage to the peritoneum significantly increased, accompanied by elevated TLR4 expression (17). These findings imply that M1 macrophages play a crucial role as mediators of peritoneal fibrosis.

In conclusion, we propose that during the development of peritoneal fibrosis, macrophages predominantly polarize into the M1 subtype during the initial phase of chronic inflammation. In contrast, a greater presence of polarized M2 macrophages is observed in the subsequent anti-inflammatory phase associated with tissue repair and fibrosis.

## Mechanisms of macrophages involvement in PD-induced peritoneal fibrosis

**Figure 2:**
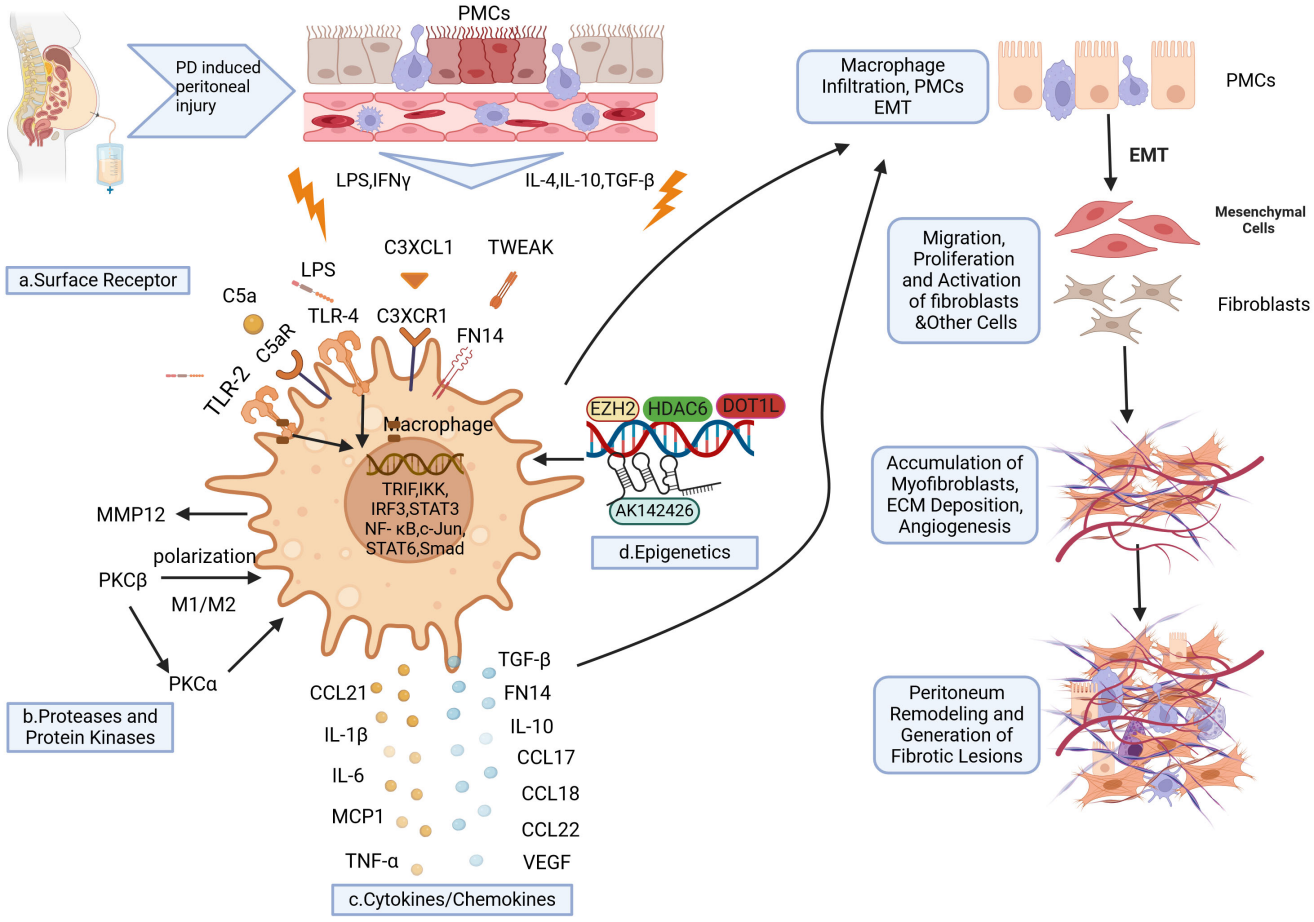
Schematic representation of macrophages implicated in the development of PD-induced peritoneal fibrosis. Long-term PD induces peritoneal injury, leading to the activation of local tissue resident macrophages and the subsequent release of various cytokines. This further promotes the recruitment of circulating macrophages to the injury site. Under cytokine stimulation, macrophages undergo polarization into various phenotypes which express specific surface receptors **(A)**. Upon binding with their respective ligands, these receptors activate transcription factors and elements that promote the expression of proinflammatory and profibrogenic factors **(C)**. Consequently, this activates peritoneal mesothelial cells (PMCs) to undergo epithelial-mesenchymal transition (EMT), thereby increasing local macrophage infiltration, myofibroblast proliferation, extracellular matrix (ECM) deposition, and neovascularization. Ultimately, these processes contribute to peritoneal fibrosis. Additionally, proteolytic enzymes and protein kinases **(B)** are also involved in the polarization of macrophages towards the M1/M2 phenotype, which further exacerbates peritoneal inflammation and fibrosis. Furthermore, epigenetic mechanisms **(D)** such as histone acetyltransferase activity, histone methyltransferase activity, and long non-coding RNAs (lncRNAs) are implicated in regulating macrophage polarization and enhancing profibrogenic factor expression during PD-induced peritoneal fibrosis. Created with BioRender.com (accessed on April 8th, 2024).

Although the involvement of macrophages in tissue and organ fibrosis has been extensively documented, there is a paucity of research on the role of macrophages in PD-associated peritoneal fibrosis, and the underlying mechanism remains incompletely understood. Given the heterogeneity and plasticity of peritoneal macrophages, as well as their diverse sources, unraveling their intricate role and interactions with peritoneal mesothelial cells poses significant challenges. This section intends to summarize the role of peritoneal macrophages in fibrosis induced by PD, drawing on findings from the last ten years (**Figure 2**).

### Surface receptor-dependent mechanisms

Cell surface receptors, which are proteins embedded in cell membranes, are essential for regulating cellular responses to environmental cues and enabling signal transduction among cells. Cytokines or signaling proteins bind to their corresponding surface receptors, leading to downstream pathway alterations that regulate various processes, including cell proliferation, migration, phagocytosis, and cytokine production. Recent research has demonstrated the role of particular cell surface receptors in peritoneal fibrosis by modulating macrophage responses.

TLRs are a diverse family of receptors found on cell membranes or in cytoplasms of different immune cells, such as macrophages, natural killer cells, lymphocytes, endothelial and epithelial cells, and fibroblasts (71). TLR2 and TLR4 represent some of the initial immune responses employed by the host to combat infections, serving a crucial function in identifying pathogens. TLR4 is predominantly expressed on macrophages and serves as a critical inflammatory modulator. Studies have indicated that during PMC EMT, TLR4 signaling dependent on TRIF is triggered by the upregulation of transcription factors TRIF, IRF3, and IKK in M1 macrophages (64, 72). Activation of the TLR4 signaling pathway promotes synthesis of TGF-β and participates in post-inflammatory fibrosis formation (73, 74). In mice depleted of macrophages, peritoneal fibrosis was completely inhibited. However, after reperfusion with M1 macrophages, there was significant damage to peritoneal structure/function along with aggravated peritoneal fibrosis due to increased expression levels of TLR4 (17). Raby et al. (75) identified the expression of TLR2, TLR4, and C5aR in peritoneal macrophages and mesothelial cells, which are essential in facilitating pro-inflammatory and pro-fibrotic responses to bacterial infections. Additionally, the use of antibody blockade targeting TLR2, TLR4, or C5aR showed considerable inhibitory effects on the bacteria-induced release of pro-fibrotic and pro-inflammatory mediators by peritoneal leukocytes in cases of infection-related peritoneal fibrosis. To sum up, TLR2 and TLR4 have been identified as promising therapeutic targets for peritoneal fibrosis linked to PD, underscoring their importance in this area of research.

The tumor necrosis factor-like apoptosis weak inducers (TWEAK, TNFSF12), members of the structurally related cytokine TNF superfamily, have the ability to regulate apoptosis, inflammation, proliferation, and angiogenesis (76, 77). Fibroblast growth factor-induced 14 (Fn14) serves as a functional receptor for TWEAK, which is significantly upregulated and plays an essential role in tissue injury, repair, and remodeling processes (78). Sanz et al. (79) identified a rise in sTWEAK levels within the peritoneal effluents of patients experiencing PD peritonitis, which was associated with the count of peritoneal macrophages. Both mesothelial cells and macrophages express Fn14 receptors, making them potential targets for TWEAK. Peritoneal biopsy suggests that TWEAK/Fn14 may have detrimental effects on mesothelial injury, as well as peritoneal inflammation and fibrosis. Injection of TWEAK into the peritoneal cavity of mice induced an upregulation in MCP-1 expression, along with Fn14 and CCL21, in both peritoneal effluents and mesothelial cells, leading to increased recruitment of macrophages in subcutaneous tissue and resulting in peritoneal inflammation and fibrosis. Overall, these results indicate that the TWEAK/Fn14 pathway might play a role in inflammatory and fibrotic damage during peritonitis and PD by facilitating macrophage recruitment. The future necessitates further investigation into the modulation of peritoneal macrophages through inhibition of the TWEAK/Fn14 pathway, with the aim of enhancing peritoneal fibrosis management.

The fractalkine receptor CX3CR1, a surface protein found on leukocytes, is highly expressed in monocytes and macrophages (80). Besides its function in monocyte adhesion and recruitment, CX3CR1 also serves as a marker for TGF-β, a pro-fibrotic cytokine (81). Studies conducted in other organs and tissues have confirmed that interactions between CX3CR1 and CX3CL1 can promote fibrosis, whereas blocking CX3CR1 with medications can lower TGF-β expression in the kidney (81–83). However, CX3CR1 has also been reported to exhibit anti-fibrotic effects (84, 85). These different outcomes may be attributed to variations in local tissue expression levels of CX3CL1 and sex differences in receptor regulation (86). Interestingly, a recent study utilized flow cytometry and global transcriptome techniques to identify high levels of CX3CR1 expression in PD effluent (55). In both patients with PD and mouse models of PD, mesothelial cells were found to express CX3CL1, while peritoneal wall macrophages predominantly expressed CX3CR1. Interactions between macrophages and mesothelial cells lead to increased expression of CX3CL1 by mesothelial cells through the action of macrophage-expressed IL-1b and enhanced TGF-β production by mesothelial cells. This promotes dialysate-induced peritoneal fibrosis. Conversely, TGF-β promotes the expression of CX3CR1 on macrophages, thereby forming a pro-fibrotic feedback loop (87). Focusing on the interactions between CXCR and CXCL is anticipated to offer new treatment possibilities for peritoneal fibrosis in individuals with PD.

### Cytokines/chemokines

Circulating monocytes are drawn to the area of peritoneal injury following stimulation, where they undergo differentiation into either proinflammatory/cytotoxic (M1) or anti-inflammatory/wound healing (M2) macrophage subsets. Polarized macrophages secrete a plethora of cytokines/chemokines that contribute to peritoneal inflammatory injury, tissue repair, fibrosis, and angiogenesis. Additionally, these secreted factors further enhance the recruitment and polarization of macrophages.

Recent investigations have revealed that classical cytokines derived from peritoneal macrophages, such as IL-6 and IL-10, are pivotal in the pathogenesis of peritoneal inflammatory damage and fibrosis through intricate signaling pathways. In particular, IL-6, mainly produced by M1 macrophages, is identified as a key pro-inflammatory factor in the peritoneal cavity of patients receiving PD (27). In an inflammatory milieu driven by TGF-β1, heightened secretion of IL-6 can induce polarization of macrophages into a M2 phenotype, thereby contributing to the development of PD-induced peritoneal fibrosis (88). Yang et al. (89) showed that the stimulation of the JAK/STAT3 signaling pathway by IL-6 along with soluble IL-6 receptor (sIL-6R) can increase VEGF levels in human peritoneal mesothelial cells (HPMCs), thereby promoting peritoneal neovascularization. Furthermore, stimulation of HPMCs with the IL-6/sIL-6R complex induces the EMT process through the transcriptional activator 3 (STAT3) pathway (90). In mouse models, intraperitoneal injection of high-glucose dialysate triggers phosphorylated activation of STAT3, resulting in macrophage infiltration, peritoneal fibrosis, and angiogenesis. Blocking IL-6/sIL-6R signaling can prevent these alterations in the peritoneum. Additionally, levels of IL-6, VEGF, and angiopoietin-2 are elevated in PD effluents from PD patients (90). In contrast, IL-10 secreted by M2 macrophages is a versatile cytokine known for its strong anti-inflammatory effects (91). Growing evidence suggests that IL-10 can significantly suppress the production of several pro-inflammatory mediators, such as TNF-α, IL-1, and IL-6, under diverse pathological conditions (92). Furthermore, research has shown that IL-10 can effectively slow down the fibrosis process in various organs and tissues, including the heart, lungs, liver, and kidneys (93–97). Previous investigations have confirmed that peritoneal IL-10 may originate from tissue resident macrophages and exert regulatory effects on antigen-presenting cell recruitment, as well as inflammatory cell infiltration (98). Onishi et al. (99) validated a significant reduction in fibrous peritoneal thickening in rats overexpressing IL-10 with peritoneal fibrosis induced by methylacetalized (MGO). Additionally, overexpression of IL-10 markedly inhibits macrophage infiltration, along with the expression of TNF-a, IL-1β, IL-6, TGF-β1, Snail transcription factor gene (Snail), and MMP2 genes, while also suppressing mesenchymal cells proliferation. Consequently, peritoneal fibrosis is significantly alleviated.

Chemokines are essential in the context of autoimmune diseases, inflammation, and tissue fibrosis, positioning them as a central focus for both researchers and clinical treatments. Within this group of chemokines, the C-C motif chemokine ligand (CCL) family, particularly CCL1, CCL2, CCL17, CCL18, CCL22, and CCL24, are closely associated with macrophage polarization and contribute to induction of inflammatory and fibrotic damage. MCP-1/CCL2 is a well-known monocyte chemokine produced by M1 macrophages that facilitates the recruitment of monocyte-derived macrophages into injured tissues while promoting local inflammatory responses (100). Clinical evidence suggests that MCP-1/CCL2 could serve as a biomarker for predicting renal fibrosis and deterioration in function (101, 102). Furthermore, animal experimental studies have utilized MCP-1/CCL2 as a therapeutic target to improve preclinical kidney disease outcomes (103–105). Following polarization, M2 macrophages are capable of producing elevated amounts of IL-10, TGF-β, CCL1, CCL17, CCL18, CCL22, and CCL24. Their primary functions encompass anti-inflammatory responses, tissue regeneration and repair processes, angiogenesis promotion, as well as immune regulation (106). CCL17 is a ligand for the chemokine receptor type 4 (CCR4) in the C-C chemokine family that was initially found to be highly expressed in the thymus and act as a chemoattractant for T cells (107). M2 macrophages are capable of secreting CCL17, which activates fibroblasts, lymphocytes, and macrophages through a signaling pathway involving interaction with CCR4 (108, 109). In a mouse model of peritoneal fibrosis induced by sodium hypochlorite exposure (16), a notable rise in the expression levels of chemokines CCL17 and CCL22 was observed in peritoneal macrophages two days after the injury. This upregulation further stimulated collagen production along with fibroblast migration. Notably, administration of an anti-CCL17 antibody resulted in a substantial reduction in peritoneal myoblast infiltration, macrophage accumulation, and fibrotic changes. Similarly, AMAC-1/CCL18 is a chemokine specific to M2 macrophages that is also known as a lung and activation regulatory chemokine. In PD patients, elevated levels of CCL18 in effluents are linked to peritoneal dysfunction and fibrosis. This suggests that peritoneal M2 macrophages may play a role in the development of peritoneal fibrosis by facilitating fibroblast proliferation and enhancing CCL18 production (67). A different study validated that elevated levels of CCL18 were found in the effluents from patients undergoing long-term PD, which was associated with a compromised peritoneal transport status and the length of time on PD (110). Consequently, CCL, a chemokine produced by macrophages, is essential in triggering peritoneal inflammation, promoting angiogenesis, and stimulating fibroblast proliferation, as well as the subsequent development of long-term PD-induced peritoneal fibrosis.

### Proteases and protein kinases-related mechanisms

Proteases are enzymes that catalyze the hydrolysis of proteins, breaking them down into smaller peptides or amino acids. Protein kinases, on the other hand, are enzymes that catalyze protein phosphorylation and regulate the final modification of proteins. Proteases and protein kinases are both vital in numerous cellular functions, including the degradation and restoration of proteins, modulation of cell signaling pathways, activation of enzyme precursors, and control of apoptosis. Recruitment, polarization, and secretion of macrophages require the participation of various signaling proteins. Therefore, proteases and protein kinases are either present or absent depending on their specific functions. This section will review the roles of and mechanisms by which proteases and protein kinases regulate macrophages during PD-induced peritoneal fibrosis.

MMPs are calcium- and zinc-dependent proteolytic enzymes that can be secreted by macrophages (111). The main role of MMPs is to break down the extracellular matrix (ECM), which is essential for processes such as cellular proliferation, growth, differentiation, migration, and communication between cells (112). Recent studies indicate that the expression of MMP-12 specifically in peritoneal macrophages from rat models with continuous exposure to glucose peritoneal dialysate. This finding suggests that PD-induced peritoneal injury involves increased expression of MMP-12 in peritoneal macrophages, underscoring the possible critical role of MMP-12 in the repair mechanisms related to peritoneal fibrosis (66). Research has demonstrated that various MMPs play a role in fibrosis following tissue and organ injury. For instance, the secretion of MMP2 and MMP9 by macrophages can promote pulmonary fibrosis in cases of idiopathic interstitial pneumonia (113). However, limited studies have been conducted on the involvement of macrophage-secreted MMPs in peritoneal dialysis-associated peritoneal fibrosis. Therefore, further investigations are warranted to elucidate the role of MMPs in macrophage-mediated regulation of peritoneal fibrosis during PD.

PKCs constitute a group of serine/threonine protein kinases, with several members widely expressed across different cells and tissues and play crucial roles in cellular function and multiple signal transduction (114). Studies have shown that PKCs serve as important signaling proteins that regulate the inflammatory cytokine response in peritoneal resident macrophages (115, 116). In a long-term mouse model of PD, upregulation and activation of peritoneal PKCα were observed, along with macrophages infiltration, EMT of peritoneal mesothelial cells, neovascularization, peritoneal fibrosis, and decreased ultrafiltration capacity (115). Administration of a PKCα blocker or gene deficiency can ameliorate the pathological changes in the peritoneum significantly by decreasing the levels of pro-inflammatory, pro-fibrotic, and pro-angiogenic mediators. In cell experiments, both PKCα blockers and PKCα deficiency inhibited MCP-1 release from mouse peritoneal mesothelial cells induced by PD while improving TGF-β1-induced EMT (115). Similarly, PKCβ expression can be observed in peritoneal macrophages of primary mice, and its upregulation is significantly induced in a high-glucose environment (70). *In vivo*, prolonged exposure to PD fluid containing high levels of glucose can lead to upregulation of peritoneal PKCβ in mice, resulting in fibrosis and neovascularization. In PKCβ gene knockout mice, all pathological alterations were significantly worsened when compared to wild-type mice. The main molecular mechanism is probably associated with the shift of peritoneal macrophages towards the pro-inflammatory M1 phenotype due to PKCβ deletion and the upregulation of PKCα in peritoneal mesial cells of mice. Furthermore, studies have also confirmed that PKCβ is involved in the M1/M2 polarization process of human peritoneal macrophages (70). In summary, these studies emphasize the importance of PKCs as an important molecular mediator of peritoneal macrophages during peritoneal fibrosis.

### Transcriptional and epigenetic mechanisms

Transcriptional expression and gene activity are significantly influenced by transcription factors and epigenetic modifications (117, 118). Increasing evidence indicates that these factors and modifications play a role in regulating the expression and activation of cytokines and signaling molecules linked to peritoneal fibrosis (119). In the context of peritonitis, NF-κB and STAT3 become activated, leading to the production of multiple proinflammatory cytokines/chemokines such as IL-6, MCP-1, TNF-α, and IL-1β (120–124). MCP1, along with other chemokines, can induce macrophage aggregation in the peritoneum, leading to the secretion of TGF-β, connective tissue growth factor (CTGF), VEGF, and MMPs, thereby promoting the progression of peritoneal fibrosis (125). Ding and his team (126) created peritoneal fibrosis in mice by injecting high glucose dialysate (HG-PDF) intraperitoneally, which led to the phosphorylation of STAT3 and an influx of macrophages into the peritoneal cavity. The inhibitor sgi-201 targeting STAT3 effectively suppresses hyperglycemia-induced peritoneal fibrosis by inhibiting angiogenesis, macrophage infiltration, and HIF-1α expression.

Enhanced zeste homolog 2 (EZH2), a histone lysine methyltransferase, is capable of catalyzing the trimethylation of histone H3 at lysine 27 (H3K27me3) and can facilitate transcriptional silencing of numerous genes, such as E-cadherin (127). Activation may also be regulated through direct methylation of certain intracellular signaling molecules, including STAT3 (128). Liu et al. (129) discovered that EZH2 was abundantly expressed in peritoneal tissue of patients suffering from PD-associated peritonitis and in dialysis effluents from individuals undergoing long-term PD. They also confirmed a positive relationship between EZH2 expression and levels of TGF-β1, VEGF, and IL-6. Similarly, increased levels of EZH2 were noted in mouse peritoneum models induced by chlorhexidine gluconate (CG) or hyperglycemic peritoneal dialysate. Moreover, inhibition therapy targeting EZH2 in both models alleviated peritoneal fibrosis and suppressed the activation of various pro-fibrotic signaling pathways, such as TGF-β1/Smad3, Notch1, EGFR, and Src, as well as phosphorylation events involving STAT3 and NF-κB. Additionally, infiltration and angiogenesis processes involving injured peritoneal lymphocytes and macrophages were reduced (129).

Histone acetylation is a post-translational modification mechanism, and the histone deacetylase (HDAC) family being crucial in modulating various physiological and pathological activities associated with acetylation (130). HDAC1, a member of class I histone acetyltransferases, has been shown to play a role in peritoneal fibrosis. It has the ability to stimulate the expression of marker genes related to MMT that are extracted from the effluent of patients receiving PD (130). Another subtype of the HDAC family, HDAC6, primarily located in the cytoplasm, affects both histone and non-histone acetylation. The expression levels of HDAC6 increase in the peritoneal cavity and dialysis fluids of individuals receiving PD. This increase shows a positive correlation with the expression of TGF-β1, IL-6, and VEGF. Inhibiting HDAC6 can suppress IL-6/STAT3 and Wnt1/β-catenin signaling, leading to a decrease in VEGF production and PD-induced angiogenesis (131). Clearly, HDAC6 is crucial in IL-6-induced peritoneal EMT, along with the proliferation and migration of mesothelial cells in the peritoneum (132). Selective inhibition of HDAC6 significantly reduces CG-induced peritoneal fibrosis progression by limiting EMT and extracellular matrix protein deposition. Research conducted both *in vivo* and *in vitro* has indicated that blocking HDAC6 significantly attenuates M2 macrophage polarization in a dose-dependently manner by inhibiting TGF-β/Smad, IL4/STAT6, and PI3K/AKT signaling (133). Therefore, targeting HDAC6 holds promise for treating peritoneal fibrosis.

Disruptor of telomeric silencing 1-like (DOT1L) is a specialized histone methyltransferase that facilitates the methylation of lysine 79 on histone H3 in mammals and is associated with tissue and organ fibrosis in many diseases (134–136). Liu et al. (137) discovered notable elevation in DOT1L expression within fibrotic peritoneal tissues from patients undergoing long-term PD, as well as in mice models. Inhibiting DOT1L can effectively reduce the transdifferentiation of mesothelial cells and phenotypic differentiation of macrophages into M2 macrophages, thereby alleviating peritoneal fibrosis. Mechanistically, DOT1L primarily participates in protein tyrosine kinase binding processes and contributes to the structural composition of the peritoneal ECM. Studies have revealed that intracellular DOT1L guides H3K79me2 upregulation for EGFR expression in mesothelial cells and and the activation of JAK3 in macrophages, while extracellular DOT1L interacts with EGFR and JAK3 to sustain activated signaling. In conclusion, DOT1L promotes the expression and activation of tyrosine kinases, specifically EGFR in mesothelial cells and JAK3 in macrophages, driving cell differentiation towards a pro-fibrotic phenotype, ultimately leading to peritoneal fibrosis. Therefore, targeting DOT1L may offer new opportunities for clinical drug development against dialysis-associated peritoneal fibrosis.

Long non-coding RNAs (lncRNAs) refer to a category of RNA molecules that are longer than 200 nucleotides and do not code for proteins. They play key roles in regulating various intracellular processes, including inflammation, proliferation, and fibrosis (138). A microarray expression profiling analysis revealed differential expression of 232 lncRNAs in fibrotic peritonea (139). Specifically, expression of AK142426 was found to be upregulated. Jiang et al. (140) observed significant upregulation of AK142426 in PD effluents from mouse models with PD-induced peritoneal fibrosis. Subsequent investigations indicated that PD treatment led to the polarization of M2 macrophages and inflammation within the PD fluid. Encouragingly, the silencing of AK142426 inhibited M2 macrophage polarization and inflammation while alleviating peritoneal fibrosis *in vivo*. Furthermore, it was discovered that AK142426 can enhance expression and activation of c-Jun through its interaction with the c-Jun protein. Overexpression of c-Jun was shown to partially reverse the inhibitory effects of sh-AK142426 on M2 macrophage activation. These studies confirm that AK142426 promotes peritoneal M2 macrophage polarization and contributes to peritoneal fibrosis by upregulating c-Jun, highlighting its potential as a potential therapeutic target for individuals affected by PD.

A growing body of studies have confirmed that transcriptional and epigenetic mechanisms are indispensable for programmed macrophage aggregation, polarization, and activation of cytokine expression. Regulation of pro-fibrotic gene expression is essential for the progression of PD-induced peritoneal fibrosis, and a deeper understanding of the transcriptional and epigenetic mechanisms that govern macrophage behavior may lead to significant advancements in preventing and treating peritoneal fibrosis. However, additional research is still required to elucidate specific mechanisms and explore potential treatment possibilities.

### Other mechanisms

The activation of NLRP3, a sensor protein for inflammasomes, triggers the assembly of oligomeric complexes that include apoptosis-associated speck-like protein (ASC) and caspase-1, collectively known as the “inflammasome.” The NLRP3 inflammasome drives caspase-1 activation by facilitating proteolytic cleavage of procaspase-1 and aiding in the transformation of the IL-1β precursor into its active variant. This process ultimately results in tissue inflammation and damage (141). Hautem et al. (142) indicated that NLRP3 inflammasomes are involved in peritoneal transport function decline and changes in peritoneal morphology during PD-associated peritonitis. In a mouse model of MGO-induced peritoneal fibrosis associated with PD, Takahashi et al. discovered that mice lacking NLRP3, ASC, and interleukin-1β (IL-1β) exhibited markedly decreased levels of inflammation-related proteins induced by PD, as well as decreased peritoneal fibrosis (143). The absence of ASC resulted in lower levels of inflammatory and fibrotic factors, as well as a reduction in macrophage infiltration. Additionally, the lack of ASC prevented the formation of MGO-induced hemorrhagic ascites, reduced fibrin deposition, and decreased the upregulation of plasminogen activator inhibitor-1. These findings suggest that endothelial NLRP3 inflammasomes participate in PD-associated peritoneal inflammation and fibrosis, providing novel perspectives on the pathogenesis of the disease.

## Novel therapeutic strategies targeting macrophages in peritoneal fibrosis

Peritoneal macrophages are crucial in the development of peritoneal fibrosis caused by PD. Peritoneal fibrosis is accompanied by persistent inflammation. The M2 phenotype of peritoneal macrophages, which has pro-fibrotic and anti-inflammatory characteristics, along with the pro-inflammatory M1 phenotype, are involved in the progression of peritoneal fibrosis. Therefore, regulating peritoneal macrophages to delay or prevent peritoneal fibrosis holds significant therapeutic potential. However, there is a paucity of research in this area to date. Literature published over the past decade describe various therapeutic approaches, encompassing cell therapy, targeted gene therapy, natural compound therapy, and biocompatible peritoneal dialysate (**Table 1**). Animal models such as MGO-, HG-PDF-, and CG-induced models have been predominantly utilized to investigate peritoneal fibrosis and have shown that interventions can effectively ameliorate this condition by inhibiting the proliferation and polarization of M2 macrophages. Additionally, the utilization of cell lines such as Raw264.7 cells for *in vitro* polarization into M2 macrophage phenotypes by M2-induced cytokines (e.g., IL-4), followed by the inhibition of this polarization using novel therapeutic approaches, has also been documented. There have also been reports indicating that therapeutic agents can attenuate the aggregation of CD68+ macrophages in the peritoneum, serving as a marker for mouse M1 macrophages in animal models of peritoneal fibrosis while alleviating peritoneal fibrosis. Additionally, some reports that we reviewed described treatments in peritoneal fibrosis models that could inhibit macrophage infiltration. However, only general markers of macrophages, such as F4/80+ and ED1+, were detected. Furthermore, certain therapeutic approaches only confirmed the regulatory effect on macrophage infiltration without establishing their impact on peritoneal fibrosis itself. In this section of the review, our focus will be on treatments that have been identified to improve peritoneal fibrosis by directly modulating macrophage polarization.

**Table 1:** Therapeutics which inhibit PD-induced peritoneal fibrosis by modulating macrophages.

Therapeutics	Macrophage phenotype	Drug route and DDS	*In vivo* model	Modeling time and dosing duration	*In vitro* cells	Ref.
Adipose-derived MSC	M2 macrophage	i.p.	MGO induced PF	10 days/once	Rat macrophage cells	(88)
SGLT2 inhibitor dapagliflozin	M2 macrophage	i.p.	HG-PDF induced PF	5 weeks/12 weeks	HPMC, MPMC, and RAW264.7 Cells	(152)
ω-3PUFA	M2 macrophage	i.p.	HG-PDF induced PF	28 days/28 days	_	(69)
Astragalus membranaceus	F4/80+ macrophage	i.p.	HG-PDF induced PF	21 days/7 days	_	(155)
Mitochonic acid-5	F4/80+ macrophages	o.g.	MGO induced PF	21 days/21 days	_	(156)
Hepatocyte growth factor	M1 and M2 macrophage	Subcutaneous (SC) osmotic pumps	MGO induced PF	14 days/14 days	_	(157)
Tubastatin A	M2 macrophage	i.p.	induced PF	21 days/21 days	Raw264.7 cells	(133)
3-Methyladenine	CD68+ macrophages	i.p.	HG-PDF or CG induced PF	PDF for 28 days and CG for 21 days	HPMCs	(158)
BLS	M1 macrophage	i.p.	BLS VS. LS	8 weeks	_	(159)
Linagliptin	F4/80+ macrophages	O.G.	MGO induced PF	3 weeks/6 weeks	_	(160)
3-DZNeP	CD68+ macrophages	i.p.	HG-PDF or CG induced PF	PDF for 28 days and CG for 21 days	HPMCs	(129)
CCL17 antibody	CD11b^int^F4/80^int^InfMϕs	i.p.	Sodium hypochlorite induced EPS	once/from day 2 to day 6	_	(16)
sh-AK142426	M2 macrophages	i.p.	HG-PDF induced PF	30 days/30 days	macrophages isolated from PD fluid	(140)
hepatocyte growth factor	Peritoneal Macrophages	i.p.	CG induced PF	3 weeks		(161)
PKC inhibitor (Go6976)	F4/80+ macrophages	i.p.	HG-PDF induced PF or PKCα–/–mice	5 weeks/5 weeks	HPMCs	(115)
spironolactone	ED1+ macrophages	gastric gavage	HG-PDF induced PF	30 days/30 days	_	(162)
IL-10	CD68+ macrophages	i.m	MGO induced PF	21 days/49 days	_	(99)

DDS, drug delivery system; MSCs, mesenchymal stem cells; ω-3PUFA, omega-3 polyunsaturated fatty acid; BLS, bicarbonate/lactate-buffered solutions; i.p., intraperitoneal injection; i.g., intragastrical administration; i.m., intramuscular-injection; MGO, methylglyoxal; PF, peritoneal fibrosis; HG-PDF, high glucose based peritoneal dialysis fuid; CG, chlorhexidine gluconate; PD, peritoneal dialysis; 3-DZNeP, 3-deazaneplanocin A.

### Therapeutic strategies targeting M2 macrophages

Mesenchymal stem cells (MSCs) have strong immunomodulatory properties and can be readily isolated and expanded *in vitro* (144, 145). In recent years, there has been extensive research on the potential for MSCs to contribute to tissue and organ repair, as well as treat neurodegenerative diseases (146). Adipose-derived mesenchymal stem cells (ADSCs), a source of MSCs, have demonstrated immunomodulatory and anti-fibrotic effects in peritoneal fibrosis (147, 148). Yang et al. (88) investigated the therapeutic impacts of ADSCs and bone marrow-derived mesenchymal stem cells (BM-MSCs) in rat models suffering from peritoneal fibrosis induced by PD. They found that ADSCs exhibited a more significant therapeutic effect on peritoneal fibrosis than BM-MSCs. Furthermore, they confirmed a positive correlation between this therapeutic effect and polarization of M2 macrophages. Additionally, *in vitro* studies demonstrated that MSCs were exposed to an inflammatory environment characterized by the presence of TGF-β1, leading to the release of IL-6 and subsequent polarization of macrophages into the M2 phenotype. Ultimately, it is concluded that ADSCs can exert an anti-peritoneal fibrosis role by regulating peritoneal macrophage polarization. Given its abundant availability and ease of acquisition, ADSCs hold considerable promise for future applications.

Prolonged exposure to elevated glucose levels is well acknowledged as a major factor leading to peritoneal fibrosis due to pathological damage in PD (43). Cell-specific sodium-dependent glucose transporters (SGLTs) possess unique glucose sensing properties and serve as the main pathway for glucose uptake by mammalian cells (149). Research has shown that SGLT1 is expressed on the apical membrane of human PMCs (150). Recent investigations have confirmed the coexistence of SGLT1 and SGLT2 in rat peritonea (151). Balzer et al. (152) applied the selective SGLT2 inhibitor dagliazin to treat mouse model of peritoneal fibrosis triggered by high glucose from PD solution. They observed significant reductions in TGF-β concentrations in PD effluents, peritoneal thickening, fibrosis, and microvascular density after dagliazin treatment, leading to improved ultrafiltration. *In vitro* experiments revealed that dagliazin promoted M2 macrophage polarization while reducing cytokine release induced by high sugar levels in human and mouse peritoneal mesoepithelial cells. In summary, through regulation of macrophage polarization, dagliazin can ameliorate structural and functional damage associated with hyperglycemic PD.

Omega-3 fatty acids, a category of polyunsaturated fatty acid, have long been recognized for their abilities to promote cardiovascular health and reduce the risk of certain chronic diseases (153). One study revealed that dialysis patients exhibited significantly lower levels of omega-3 fatty acids, leading to an increased cardiovascular risk in those with chronic kidney disease (154). Liu et al. (69) showed that prolonged intravenous delivery of omega-3 fatty acids in PD rats effectively suppressed the expression of activation-related proteins (i.e., Erg2 and IRF4) in peritoneal M2 macrophages, whereas no notable changes were seen in the levels of activation-related proteins (i.e., CD38 and IRF5) in M1 macrophages. Furthermore, omega-3 fatty acids were found to significantly decrease the levels of fibrosis-related factors such as α-SMA, TGF-β1, and VEGF, as well as ALK5 proteins, thereby ameliorating peritoneal fibrosis.

Tubastatin A (TA) acts as a selective inhibitor of HDAC6. Liu et al. (133) discovered that TA effectively inhibits the polarization of M2 macrophages, suppresses the expression of MMP2 and MMP-9, and reduces EMT, as well as ECM, protein deposition in peritoneal mesoepithelial cells by blocking the TGF-b/Smad3, PI3K/AKT, STAT3, and STAT6 pathways. Consequently, TA significantly hinders the progression of peritoneal fibrosis. *In vitro* studies indicated that both IL-4 and HG-PDF promote M2 macrophage polarization by increasing the expression of CD163 and arginase-1. However, However, TA can inhibit HDAC6 activity in a dose-dependent manner, effectively reversing M2 macrophage polarization by downregulating TGF-b/Smad, IL4/STAT6, and PI3K/AKT signaling. To summarize, blocking HDAC6 with TA may ameliorate CG-induced peritoneal fibrosis progression by abolishing M2 macrophage polarization.

Jiang et al. (140) performed a study examining the impact of lncRNA AK142426 on peritoneal fibrosis and observed significant upregulation of AK142426 in the effusions of PD mice. Knocking down AK142426 was found to inhibit PD-induced polarization of M2 macrophages and inflammation, thereby alleviating peritoneal fibrosis. Furthermore, AK142426 was found to enhance expression and activation of c-Jun by interacting with c-Jun protein. Overexpression of c-Jun partially counteracted the suppressive effects of sh-AK142426 on M2 macrophage activation and inflammation. These findings confirm that AK142426 facilitates polarization of peritoneal M2 macrophages and contributes to peritoneal fibrosis via upregulation of c-Jun, highlighting its potential as a therapeutic target for patients suffering from peritoneal fibrosis.

Hepatocyte growth factor (HGF) is a growth factor derived from mesenchymal cells that has various effects on target cells (163). Studies have demonstrated significantly higher HGF concentrations in dialysate from patients with ultrafiltration failure (164). According to Tanoue et al. (157), HGF can notably decrease the quantity of peritoneal macrophages and markers associated with M1/M2 macrophages, including CD86, CD206, and CD163 in mice with methylglyoxal-induced peritoneal fibrosis. It also reduces the levels of pro-inflammatory cytokines TGF-β, TNF-α, and IL-1β. Furthermore, HGF can markedly decrease peritoneal membrane thickness and inhibit type I/III collagen expression, as well as α-SMA levels. In another study, macrophages were employed as vehicles to intravenously express and deliver the HGF gene to mice with peritoneal fibrosis induced by chlorhexidine gluconate. This approach led to significant reductions in α-SMA/TGF-β-positive cells within the peritoneum. Additionally, transplantation of these modified macrophages resulted in reduced intersubcutaneous thickening, along with decreased type III collagen expression, while maintaining ultrafiltration function (161).

### Therapeutic strategies targeting M1 macrophages

New treatment strategies for peritoneal fibrosis, in addition to inhibiting aggregation and polarization of M2 macrophages, have been reported that target M1 macrophages, resulting in reductions in peritoneal fibrosis. Vervloet et al. (159) observed that, compared to standard lactic acid, bicarbonate/lactic acid buffer solution treatment resulted in a greater percentage of pro-inflammatory M1 macrophages within the pro-fibrotic M2 subgroup in PD mice. Liu et al. (158) showed that the autophagy inhibitor 3-MA markedly decreased infiltration of CD68-positive peritoneal macrophages, reversed EMT, and effectively prevented peritoneal fibrosis in rats. Administration of 3-deazaneplanocin A (3-DZNeP) in mouse models of CG- or HG-PDF-induced peritoneal fibrosis has been shown to mitigate peritoneal injury by reducing CD68+ macrophage invasion and angiogenesis through the inhibition of EZH2 methylation activity. Additionally, 3-DZNeP inhibits activation of multiple pro-fibrotic signaling pathways, thereby ameliorating peritoneal fibrosis (129). One study showed that overexpression of IL-10 significantly reduced infiltration of CD68+ peritoneal macrophages and thickening of the fibrous peritoneum in rats, resulting in notable relief from peritoneal fibrosis (99).

### Therapeutic strategies targeting non-categorized macrophages

Besides M1 and M2 macrophages, non-categorized macrophages, including F4/80+ and ED1+ macrophages have been the subject of several other studies related to peritoneal fibrosis. In the CG-induced mouse model of peritoneal fibrosis, there was an accumulation of CD11b^int^F4/80^int^ inflammatory macrophages (InfMϕs), while injection of anti-CCL17 antibodies significantly reduced InfMϕs, myofibroblasts, and peritoneal fibrosis in mice (16). Recent studies have indicated that astragalus extract can inhibit recruitment and activation of F4/80-positive mononuclear/macrophage cells in PD rat models, ultimately alleviating peritoneal fibrosis (155). Mitochondrial acid-5 (MA-5), an indole-3-acetic acid derivative, is a newly discovered drug that targets mitochondria. Torigoe et al. (156) showed that MA-5 treatment may improve peritoneal fibrosis by inhibiting F4/80-positive macrophages and oxidative stress, which in turn helps to restore mitochondrial function. Shushakova et al. (115) used the PKC inhibitor Go6976 or a PKCα gene defect to block the biological activity of PKCα in PD mice, resulting in alleviation of F4/80-positive macrophage invasion, peritoneal fibrosis, and neovascularization. In mice suffering from peritoneal fibrosis induced by MGO, administration of ligagliptin, which is a dipeptidyl peptidase-4 (DPP-4) inhibitor, blocked the infiltration of F4/80-positive macrophages and collagen deposition while improving peritoneal function (160). Competitive inhibitors targeting aldosterone mainly bind competitively to mannose receptors. Hao et al. (162) found that spironolactone treatment in rat models induced by PD dialysate and LPS reduced infiltration of ED-1-positive macrophages in rat peritoneal tissue, as well as decreased inflammation and fibrosis.

To conclude, the existing literature indicates that macrophages are essential in hindering the treatment of peritoneal fibrosis caused by PD. Some therapeutic studies have directly shown that amelioration of peritoneal fibrosis can be achieved through regulating macrophage polarization, while other reports have only observed a reduction in fibrotic macrophage infiltration accompanying the improvement of peritoneal fibrosis. Additionally, research has demonstrated that peritoneal macrophages undergo polarization into various phenotypes at different stages of peritoneal dialysis-induced injury. The literature reports reveal significant *in vivo* variability and variations in modeling time among the animal models employed to investigate peritoneal dialysis injury. According to research reports, the duration required for modeling therapeutic strategies targeting M1 macrophages or F4/80 positive macrophages typically does not exceed three weeks; however, establishing animal models for therapies primarily directed at M2 macrophages generally necessitates a longer timeframe. However, it remains uncertain whether inhibiting phenotypic changes in fibrotic macrophages contributes to a reduction in peritoneal fibrosis. Many studies investigating the molecular mechanism underlying macrophage-mediated peritoneal fibrosis have not explored the direct impact of cytokines on macrophages, while some recent drug studies on peritoneal fibrosis have failed to consider the polarization of macrophages. Consequently, future investigations into pharmacological interventions aimed at inhibiting peritoneal fibrosis through the regulation of peritoneal macrophages should prioritize the selection of appropriate methodologies and timelines for animal model development. Additionally, there is a pressing need for further research to focus on therapeutic agents that directly modulate macrophage polarization in order to enhance outcomes related to peritoneal fibrosis.

## Conclusions

In recent decades, our comprehension of the biological functions of macrophages in fibrosis has been significantly advanced through the application of innovative research methodologies. This article presents thorough review of the molecular mechanisms and therapeutic strategies involving peritoneal macrophages in peritoneal fibrosis. The involvement of different macrophage subpopulations found in effluents from PD patients, along with their significant roles in the development of peritoneal fibrosis, has been demonstrated both in human cases and mouse models. A plethora of studies have underscored the impact of cytokines, transcription factors, and epigenetic modifications on macrophage activity during peritoneal fibrosis, rendering them potential therapeutic targets for the management of this condition and offering valuable insights for future research endeavors. However, there is limited research on the mechanisms underlying phenotypic reprogramming of macrophages during PD and their association with peritoneal fibrosis. Therefore, further studies are warranted in the future to elucidate the properties of various aspects related to macrophage involvement in peritoneal fibrosis, including macrophage half-lives, tissue transport capabilities, activation patterns, and plasticity potential, as well as their dynamic interactions within the PD microenvironment. Moreover, the dynamic interaction of macrophages with other peritoneal cells is crucial for stimulating macrophage heterogeneity and promoting fiber formation. A variety of innovative methods, including spatial transcriptomics, protein expression analysis, single-cell RNA sequencing, and automated approaches integrated with high-throughput drug screening, could enhance research into the mechanisms driving macrophage phenotypic reprogramming and aid in identifying molecules related to anti-fibrotic macrophage phenotypes. The incorporation of these novel research findings and methodologies can facilitate a more profound comprehension of the mechanism underlying macrophage participation in peritoneal fibrosis, thereby fostering further exploration into innovative therapeutic approaches for this condition.
