# Supplementary figures and images for: Mechanisms underlying the involvement of peritoneal macrophages in the pathogenesis and novel therapeutic strategies for dialysis-induced peritoneal fibrosis
Source: Front Immunol. 2024 Dec 19;15:1507265. doi: 10.3389/fimmu.2024.1507265 (PMC11693514; doi:10.3389/fimmu.2024.1507265)

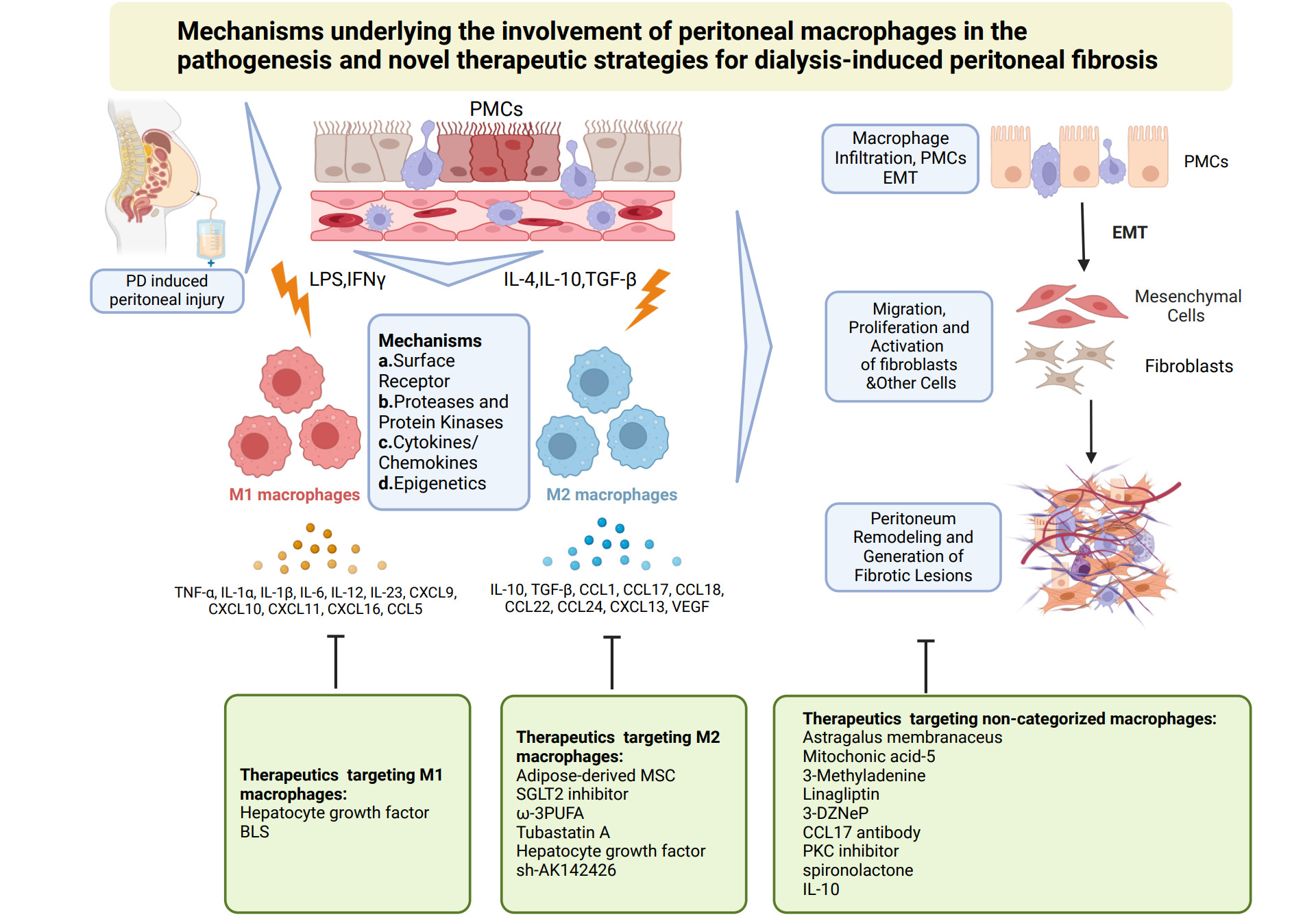

Supplement: Supplementary file 1 [file Image1.jpeg]
